# Juvenile Nasopharyngeal Angiofibroma With Skull Base Involvement: A Radiological and Clinical Insight Into a Classic Adolescent Presentation

**DOI:** 10.7759/cureus.91191

**Published:** 2025-08-28

**Authors:** Zineb Labbi, Oumaima Boukhlouf, Meriem Fikri, Mohammed Jiddane, Firdaous Touarsa

**Affiliations:** 1 Department of Diagnostic Radiology, Ibn Sina University Hospital, Rabat, MAR; 2 Department of Maxillofacial Surgery, Ibn Sina University Hospital, Mohammed V University, Rabat, MAR; 3 Department of Neuroradiology, Specialty Hospital, Rabat, MAR; 4 Department of Radiology, Ibn Sina University Hospital, Rabat, MAR

**Keywords:** adolescent nasal mass, juvenile nasopharyngeal angiofibroma, mri, skull base erosion, sphenopalatine foramen

## Abstract

Juvenile nasopharyngeal angiofibroma (JNA) represents a rare, histologically benign yet locally aggressive vascular tumor that predominantly arises in adolescent males. Given its propensity for invasive growth and involvement of critical anatomical structures, precise radiological evaluation is vital prior to any intervention. We present the case of a 13-year-old male who experienced progressive bilateral nasal blockage and recurrent epistaxis predominantly on the left side. Magnetic resonance imaging (MRI) identified a vividly enhancing lesion centered at the left sphenopalatine foramen, with extensions into adjacent sinuses, the pterygopalatine fossa, infratemporal fossa, and early skull base erosion. This case highlights the critical diagnostic and staging role of MRI in JNA, illustrating how characteristic imaging findings inform diagnosis, classification, and surgical planning.

## Introduction

Juvenile nasopharyngeal angiofibroma (JNA) is a rare, histologically benign yet locally aggressive vascular tumor, accounting for only 0.05%-0.5% of head and neck neoplasms. It predominantly affects adolescent males between the ages of 10 and 20 [1]. Although non-malignant, JNA arises from the region of the sphenopalatine foramen and demonstrates infiltrative growth patterns, often spreading along anatomical foramina and low-resistance planes into adjacent areas such as the paranasal sinuses, infratemporal fossa, and skull base, with occasional intracranial extension [1,2].

The classic clinical triad - progressive nasal obstruction, recurrent epistaxis, and a nasopharyngeal mass - is highly suggestive of JNA and should raise immediate clinical suspicion. However, definitive diagnosis relies on imaging, as biopsy is contraindicated in most cases due to the high risk of hemorrhage [1,3].

Cross-sectional imaging plays a central role in diagnosis and staging. Magnetic resonance imaging (MRI) is the preferred modality for assessing tumor extent, vascularity, and soft tissue infiltration, while computed tomography (CT) offers complementary detail in evaluating bony remodeling, foraminal widening, and the Holman-Miller sign [1,4]. It is also essential to distinguish JNA from other rare but important differential diagnoses, including rhabdomyosarcoma, antrochoanal polyps, lymphoma, nasopharyngeal carcinoma, and inverted papilloma.

This case report presents an advanced-stage JNA in a 13-year-old boy, emphasizing the hallmark imaging characteristics, relevance of Radkowski staging, and current considerations for surgical planning and management.

## Case presentation

A 13-year-old previously well male presented with a three-month history of gradually worsening bilateral nasal obstruction, more pronounced on the left side, accompanied by intermittent left-sided epistaxis. There were no associated visual complaints, headaches, or signs of cranial nerve dysfunction. Physical examination revealed congestion within the left nasal cavity, and posterior rhinoscopy identified a reddish, polypoid mass occupying the left choana. Given the patient's demographic profile and presenting symptoms, a clinical suspicion of JNA was raised. To mitigate bleeding risk, an MRI of the brain and facial bones was performed prior to any biopsy [1,3]. The MRI protocol included standard axial, coronal, and sagittal planes utilizing T1-weighted, T2-weighted, diffusion-weighted imaging (DWI), FLAIR, and post-contrast fat-suppressed T1 sequences.

**Figure 1:**
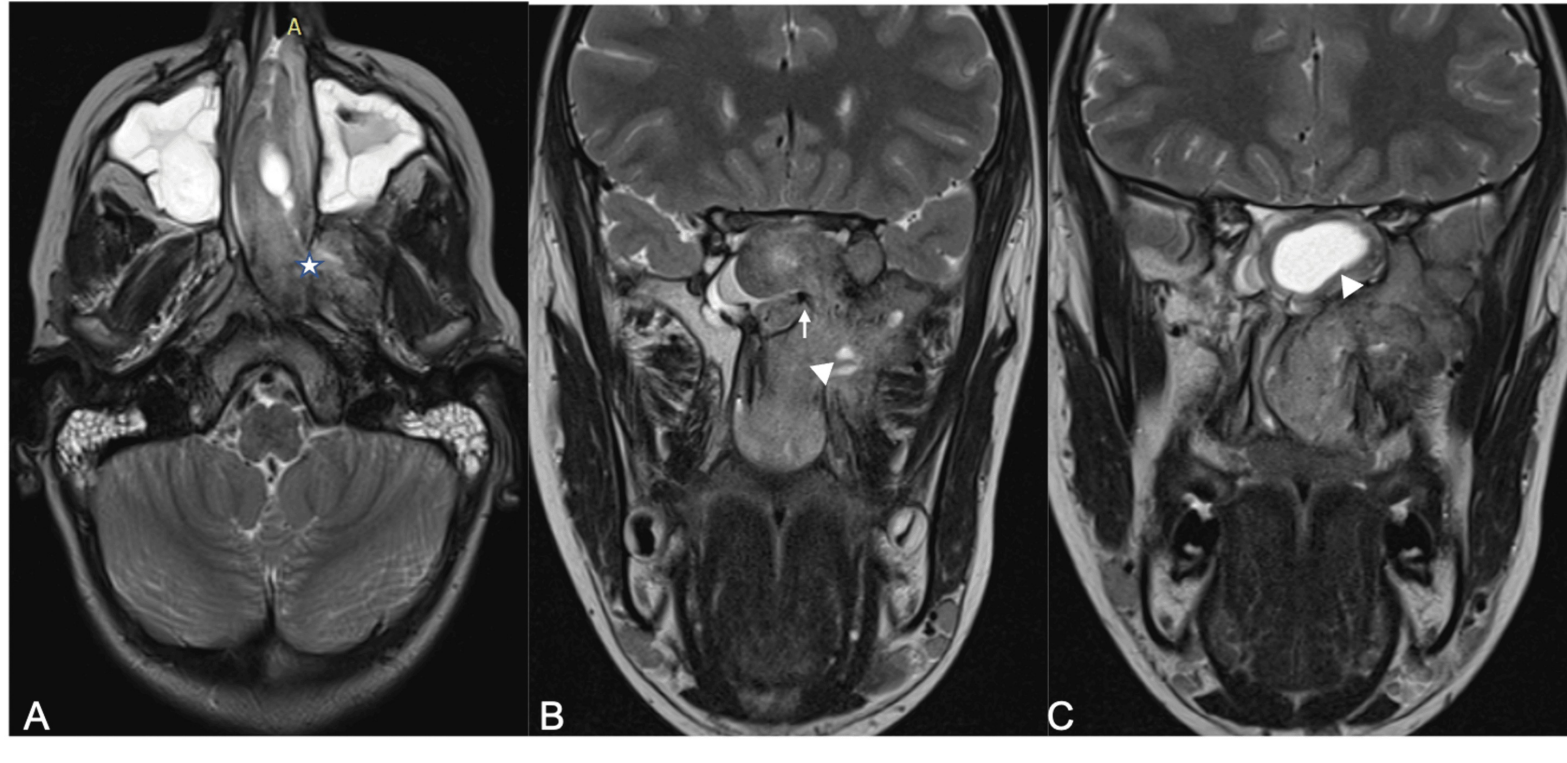
MRI findings of juvenile nasopharyngeal angiofibroma (JNA). Axial (A) and coronal (B, C) T2-weighted images. JNA appears as a mass typically arising from the sphenopalatine foramen, which appears enlarged (asterisk). It classically shows intermediate-to-high heterogeneous signal with serpentine flow voids (arrow) and degenerative cystic components (arrow head).

**Figure 2:**
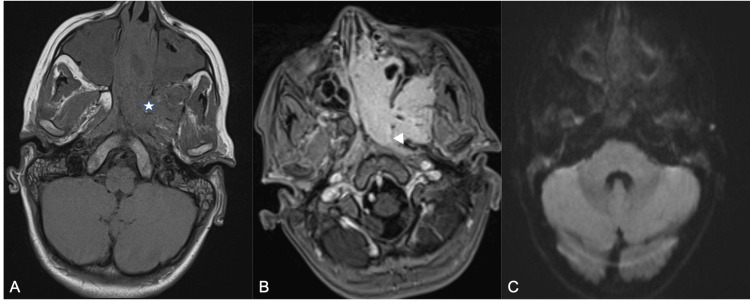
Contrast-enhanced MRI highlighting local invasion of adjacent structures with no restriction on DWI imaging. At axial non-contrast T1-weighted images (A). JNA is predominantly isointense arrising from the spheno-palatine foramen (asterisk), with intense and heterogeneous enhancement after contrast injection (B) and in this case invasion of the adjacent pterygoid muscle (arrow head). At axial DWI (C), JNA does not present a high signal.

Imaging revealed a lobulated, well-defined soft tissue mass centered in the region of the left pterygo-maxillary fissure and the sphenopalatine foramen, which appeared to be enlarged, demonstrating intermediate to high signal intensity on T2 and intermediate signal on T1 with cystic components and flow voids consistent with hypervascularity (Figures 1-2). 

Post-contrast sequences showed intense, homogeneous enhancement, characteristic of a hypervascular lesion [1,3] (Figure 2). There was no evidence of necrosis, hemorrhage, or restricted diffusion, findings in keeping with the behavior of a benign, hypervascular tumor lacking aggressive cellular features [4]. Medially, the tumor expanded into the left nasal cavity, displacing the nasal septum contralaterally and causing significant obstruction. This extension into the nasal cavity and pterygopalatine fossa is well-demonstrated in Figure 1.

Laterally, the tumor infiltrated the pterygopalatine fossa and obliterated the pterygomaxillary fissure, with further extension into the adjacent pterygoid muscles and infratemporal space, as highlighted on the axial post-contrast sequence (Figure 2).

**Figure 3:**
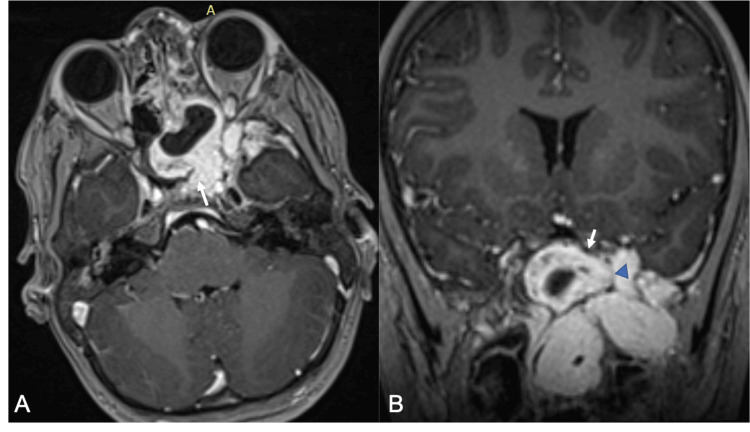
Post-contrast imaging highlighting superior extension with erosion of the sphenoid sinus and intracranial extension. Axial (A) and coronal (B) post gadolinium T1-weighted images showing superior tumor spread beyond the sphenoid sinus into the planum sphenoidale (arrow) with a small left extra-axial anterior temporal component (arrow head).

Superiorly, the lesion extended into the sphenoid and ethmoid sinuses, with early erosive changes visible in the walls of the sphenoid sinus and the greater wing of the sphenoid bone [1,3], making its way into the planum sphenoidale with a small left extra-axial anterior temporal component, all of which are consistent with intracranial extension (Figure 3).

Posterior extension involved the nasopharynx, choanae, torus tubarius, and the Eustachian tube orifice, anatomical features that could contribute to conductive hearing symptoms [1,3].

Secondary findings included secretions within the paranasal sinuses and middle ear cavities, consistent with bilateral retention-type sinusitis and otomastoiditis, likely due to obstructed drainage pathways rather than direct tumor invasion [3]. Notably, there was no evidence of orbital invasion or perineural spread. Based on the imaging features and anatomical spread, these findings are consistent with Radkowski stage IIIA (Table 1) or Fisch IIIB - reflecting advanced local invasion with skull base involvement but sparing the intracranial compartment [2,3].

**Table 1:** Radkowski classification. Sources: Refs [2,3]

Stage	Description
Ia	Limited to nose and/or nasopharynx.
Ib	Same as Ia, but with extension into one or more paranasal sinuses.
IIa	Minimal extension through the sphenopalatine foramen, into and including a minimal part of the medial-most part of the pterygomaxillary fossa.
IIb	Full occupation of the pterygomaxillary fossa, anterior displacement of the posterior wall of the maxillary antrum. Lateral and/or anterior displacement of branches of the maxillary artery. Superior extension may occur, eroding orbital bones.
IIc	Extension through the pterygomaxillary fossa into the cheek and temporal fossa, or posterior to the pterygoid plates.
IIIa	Erosion of the skull base with minimal intracranial extension.
IIIb	Erosion of the skull base with extensive intracranial extension with or without cavernous sinus invasion.

To further evaluate and confirm the vascular nature of the lesion, digital subtraction angiography (DSA) was performed as part of the first-stage treatment protocol, enabling preoperative embolization of the tumor’s feeding vessels. This significantly reduced intraoperative bleeding risk and optimized conditions for surgical resection.

## Discussion

Originating near the sphenopalatine foramen in the posterior nasal cavity, juvenile nasopharyngeal angiofibroma (JNA) is associated with regions of rich vascularity and embryological activity [1,5]. Though rare, its presentation follows a well-established clinical pattern, requiring prompt recognition due to its invasive potential and risk of significant bleeding [1,3].

The classic clinical triad of nasal obstruction, epistaxis, and a nasopharyngeal mass strongly suggests JNA. Nevertheless, diagnosis hinges on imaging, as biopsy remains contraindicated in most cases due to the risk of hemorrhage [1,4]. MRI remains the imaging modality of choice for delineating soft tissue involvement and subtle tumor extensions, while CT - although not performed in this case - offers complementary value, especially in evaluating bony changes, such as the Holman-Miller sign, foraminal widening, and erosion of the pterygoid plates, which can guide surgical planning [1,3].

In this case, MRI revealed a mass measuring approximately 4.5 × 3.8 × 3.2 cm, isointense on both T1- and T2-weighted sequences, with intense and homogeneous post-contrast enhancement-findings consistent with the tumor’s hypervascularity. The lesion demonstrated characteristic spread through the pterygopalatine fossa and infratemporal region, along with early skull base erosion. Importantly, there was no intracranial extension.

These features correspond to Radkowski stage IIIA (Table 1), denoting skull base involvement without intracranial invasion - a crucial consideration when determining the surgical approach [2,3].

Surgical excision remains the definitive treatment, typically preceded by preoperative embolization to minimize intraoperative blood loss [3,6]. While endoscopic resection is favored for early-stage tumors, advanced lesions infiltrating the infratemporal fossa or skull base, as in this case, may require a combined endoscopic and open approach (e.g., transpalatal, transmaxillary, or midfacial degloving techniques) [3]. Complete resection is paramount, given recurrence rates exceeding 30% with incomplete removal [3,6]. Recent advancements include the use of preoperative embolization with agents such as polyvinyl alcohol or Onyx, the integration of intraoperative navigation and image-guided surgery, and experimental approaches including anti-angiogenic agents, hormonal therapies, and stereotactic radiosurgery for residual disease [3,6].

While the MRI features in this case were classic for JNA, it is essential to consider potential differential diagnoses to enhance diagnostic accuracy. Entities that can mimic JNA on imaging include rhabdomyosarcoma, childhood nasopharyngeal carcinoma, antrochoanal polyp, lymphoma, and inverted papilloma. Rhabdomyosarcomas often present with infiltrative soft tissue masses, heterogeneous enhancement, and associated bone destruction, frequently showing restricted diffusion and nodal involvement [7]. Childhood nasopharyngeal carcinoma typically demonstrates intermediate enhancement, often accompanied by cervical lymphadenopathy and skull base erosion, and may show FDG uptake on PET-CT [8]. Antrochoanal polyps are usually unilateral and cystic and arise from the maxillary sinus, extending into the nasopharynx without signs of aggressive vascularity or bone invasion [9]. Lymphomas tend to appear as homogeneous soft tissue masses with mild-to-moderate enhancement and diffusion restriction and may be associated with systemic symptoms and nodal disease [10]. Inverted papillomas, although more common in middle-aged adults, can rarely occur in adolescents and manifest as unilateral nasal masses with a cerebriform pattern on MRI, particularly on T2-weighted and post-contrast images, which can help differentiate them from hypervascular tumors such as JNA [11]. The inclusion of these differentials provides a more comprehensive educational perspective, helping differentiate among lesions with overlapping clinical and imaging features.

## Conclusions

JNA, though uncommon, should remain a key diagnostic consideration in adolescent males presenting with unilateral nasal obstruction and recurrent epistaxis. This case highlights the central role of MRI in identifying classical imaging features, accurately staging the tumor, and guiding surgical planning. In addition, angiographic assessment and preoperative embolization, when feasible, are valuable adjuncts to reduce intraoperative bleeding and enhance surgical safety. Early multidisciplinary collaboration among radiologists, ENT surgeons, and interventional radiologists is essential for achieving optimal patient outcomes.
